# Feeding on the Fruit Waste Orange Bagasse Modifies Immature Protein Content, Body Weight, Scent Bouquet Composition, and Copula Duration in Males of a Tephritid Frugivorous Fly

**DOI:** 10.3390/biology12050739

**Published:** 2023-05-19

**Authors:** Carlos Pascacio-Villafán, Larissa Guillén, Alma Altúzar-Molina, Julio A. Tellez-Mora, Enedina Cruz-Hernández, Martín Aluja

**Affiliations:** 1 Red de Manejo Biorracional de Plagas y Vectores, Clúster Científico y Tecnológico BioMimic®, Instituto de Ecología, A.C., Xalapa 91073, Veracruz, Mexico; larissa.guillen@inecol.mx (L.G.); alma.altuzar@inecol.mx (A.A.-M.); enecruzhdez@gmail.com (E.C.-H.); martin.aluja@inecol.mx (M.A.); 2 Facultad de Ciencias Agrícolas, Universidad Veracruzana, Xalapa 91090, Veracruz, Mexico; t_juv@hotmail.com

**Keywords:** *Anastrepha ludens*, Diptera rearing, fruit waste management, sexual pheromones, larval protein, feeding behavior, saprophagy, orange bagasse, copulation

## Abstract

**Simple Summary:**

We successfully reared the frugivorous fruit fly pest, *Anastrepha ludens*, in cost-free fruit waste (orange bagasse), which produced individuals with lower body nutritional content and reduced body weight, and adult males with a highly chemically diverse bouquet of odors, containing eight additional compounds when compared to adults from an artificial diet. Adults from the orange bagasse diet were sexually competitive, but their copulations were significantly shorter than the copulations of males from the artificial diet and males from the wild host plant White Sapote. From a theoretical perspective, our results are relevant because they represent a system that will allow us to study insect evolution from saprophagy to frugivory, as here we grew larvae in a proxy of an ancestral medium. From an applied perspective, our findings are relevant because understanding the role of odors in sexual behavior is key to improving biorational pest control strategies. In addition, mass-rearing pestiferous fruit flies cheaply is an essential component for control programs based on the Sterile Insect Technique.

**Abstract:**

*Anastrepha ludens* is a polyphagous frugivorous tephritid that infests citrus and mango. Here, we report the establishment of a laboratory colony of *A. ludens* reared on a larval medium that is a waste for the citrus industry, specifically, orange (*Citrus* × *sinensis*) fruit bagasse. After 24 generations of rearing on a nutritionally poor orange bagasse diet, pupae weighed 41.1% less than pupae from a colony reared on a nutritionally rich artificial diet. Larvae from the orange bagasse diet had 6.94% less protein content than larvae from the artificial diet, although their pupation rate was similar. Males from the orange bagasse diet produced a scent bouquet with 21 chemical compounds and were sexually competitive, but they had significantly shorter copulations when compared to males from the artificial diet and from the wild host, *Casimiroa edulis*, which had relatively simple scent bouquets. The chemical complexity in the odors of males from the orange bagasse diet might initially have attracted females to novel scent combinations, but, once in the copula, they may have been able to sense negative characteristics in males, leading them to terminate copulations soon after they began. We conclude that *A. ludens* can adjust morphological, life history, nutritional, and chemical traits when adapted to a larval environment consisting of fruit bagasse.

## 1. Introduction

Fruit flies (Diptera: Tephritidae) have a cosmopolitan distribution, and their larvae feed mainly on the fruit of a wide variety of wild and cultivated plant species [1,2,3]. For instance, the diet breadth of the Mexican fruit fly, *Anastrepha ludens* (Loew), a polyphagous pest species distributed from Southern Texas to Costa Rica, includes grapefruit (*Citrus* × *paradisi* Macfad. [Rutaceae]), oranges (*C.* × *aurantium* L., *C.* × *sinensis* L. [both Rutaceae]), mangoes (*Mangifera indica* L. [Anacardiaceae]), among other species of tropical fruit into which female flies oviposit and on which the larvae feed [4,5,6]. Tephritid fruit flies are phytophagous, but it is believed that ancestral tephritids were saprophagous and later evolved to a frugivorous larval lifestyle [1,7]. In fact, tephritid fruit fly larvae feed at the beginning of their development on green and ripe fruit when it is still attached to the tree, but as they continue to grow, the fruit begins to decompose, and larvae usually end their growth in decaying fruit [8].

The nutritional, chemical, and physical characteristics of the larval diet environment affect the expression of many biological traits of flies, such as pupation, body weight, development time, adult emergence, and the chemical composition of male sexual pheromones, among others [3,9,10,11,12,13,14]. For instance, in *A. ludens*, low levels of protein in the larval diet reduce the survival and the weight of larvae and pupae [15]. Flies may experience phenotypic plasticity and gene expression changes in response to variations in the nutritional content of the larval diet [16]. Understanding the factors that affect the expression of immature and adult traits is key to improving our theoretical and practical understanding of the biology, physiology, ecology, and rearing of insects [9,14,17,18,19].

Many laboratories and facilities around the world use artificial diets in place of fruit to maintain colonies of tephritid pests for research and/or for the application of environmentally friendly methods of pest control, such as the Sterile Insect Technique (SIT), which involves rearing massive numbers of flies [20,21]. Artificial larval diet formulations for rearing tephritids are mixtures of ingredients, including sources of nutrients (e.g., inactive dried yeast and sugar), texturizing and bulking agents (e.g., corn cob powder, coconut fiber and cane bagasse), gelling agents (e.g., agar and carrageenan), pH regulators (e.g., citric acid and hydrochloric acid), preservatives (e.g., sodium benzoate and methylparaben), and water [22,23,24]. Some of the bulking agents that have been tested or are used in tephritid larval diet formulations include low-cost by-products, such as coconut fiber in a generic diet for *A. ludens*, *Anastrepha obliqua* (Macquart), *Anastrepha serpentina* (Wiedemann), and *Anastrepha striata* Schiner [25], as well as cane bagasse in *Ceratitis capitata* (Wiedemann) diets [24,26]. Using agricultural organic wastes as insect diets is an attractive concept from sustainability and waste management perspectives [27,28], but the idea of using fruit waste as the only larval substrate for the artificial rearing of tephritid fruit flies has not been explored. 

Here, we present information on the establishment of a laboratory colony and a rearing system [17] of *A. ludens* reared on a diet based on orange (*C.* × *sinensis*) fruit bagasse (i.e., the orange residue that is discarded after juice extraction) as the only larval substrate. We present the results of a comparative study of production, life-history, morphological, nutritional, and chemical traits of *A. ludens* flies that developed on a conventional artificial diet-rearing system [29] and on an orange bagasse diet. We also report the results of a multiple-choice sexual competitiveness test with wild *A. ludens* females from the wild host White Sapote, *Casimiroa edulis* La Llave (Rutaceae), as well as males that developed in White Sapote, the artificial diet-rearing system, and the orange bagasse diet-rearing system. We choose to study orange fruit bagasse because: (i) citrus fruit (e.g., orange and grapefruit) are natural hosts of *A. ludens*; (ii) it is a substrate that is readily available all year round in Mexico; and (iii) to find a use for the millions of tons of citrus fruit waste generated each year after industrial processing for juice production [30]. Because orange bagasse has low nutritional content [31,32], our working hypothesis when we started the orange bagasse diet-rearing system was that orange bagasse sustains the development of *A. ludens* larvae, but at the cost of reduced fitness due to the nutritional deficiencies of the bagasse. Based on the “core blend” concept proposed by Aluja et al. [12], we also hypothesized that the chemical composition (i.e., the “core blend” in the context of this study) of volatiles from sexually mature male flies of *A. ludens* from a laboratory colony that has been maintained for over 120 generations on the artificial diet-rearing system would be conserved, regardless of shifts to a novel dietary environment. Finally, given that the chemical composition of the purported sexual pheromone [12] and male mating success of *A. ludens* is influenced by its larval diet environment [33], we hypothesized that a highly chemically diverse scent bouquet beyond the “core blend” in the effluvia of sexually mature male flies enhances their sexual attractiveness [12]. To validate the veracity of our hypotheses, we experimentally tested the following predictions: (i) higher survival of flies with higher body weights and shorter development times when developed on the artificial diet-rearing system than when developed on the orange bagasse diet-rearing system; (ii) higher protein content of the diet and third instar larvae in the artificial diet-rearing system than in the orange bagasse diet-rearing system; (iii) a simpler scent bouquet in sexually mature *A. ludens* males from the artificial diet compared to those from the orange bagasse diet; and (iv) shorter time to initiate sexual activity, higher mating proportion, and copulation duration in flies with a more diverse scent bouquet than in males with a simpler scent bouquet.

## 2. Materials and Methods

### 2.1. Anastrepha ludens Artificial Diet-Rearing System

The *A. ludens* laboratory colony of the Red de Manejo Biorracional de Plagas y Vectores of the Instituto de Ecología A.C., Xalapa, Veracruz, Mexico (RMBPV), was established in 1998, and, since then, it has been maintained with minor adjustments based on methods described in Aluja et al. [29]. The last introduction of wild flies (25 females and 27 males) from citrus fruit to this colony was made in 2013. In 2017, this fly colony migrated to the “Planta Piloto de Cría de Moscas de la Fruta y Parasitoides”, a new facility at the BioMimic^®^ Scientific and Technological Cluster of INECOL. Groups of ca. 3,700 adult flies (female: male ratio near to 1:1) were kept in plexiglass cages (60 cm long × 30 cm height × 30 cm wide) at 26 ± 1 °C, 60 ± 5% relative humidity (RH), as well as a 12: 12 h L: D photoperiod. Flies in cages had ad libitum access to water and food (a 3: 1 mixture of sugar: hydrolyzed protein) [29]. Female flies oviposited into circular oviposition devices with a transparent inert gel (Furcellaran) that were placed on the top of the cage [29]. Eggs were collected from the oviposition devices and washed to remove all the gel substrate. A 1 mL volume of eggs (ca. 14,000 eggs, [34]) was spread on a piece of black terylene cloth on top of moistened felt (moistened with a 0.2% *w/v* sodium benzoate solution) inside a Petri dish and incubated in a 24 h dark laboratory at 29 ± 1 °C and 70 ± 5% RH for four days until larvae began to hatch (mean hatch = 75.8%, estimated from 10 samples of 100 eggs each). The larvae were inoculated to 1.3 kg of an artificial diet in a plastic tray (32 cm long × 19 cm wide ×14 cm high) by placing a piece of black terylene cloth with eggs and newly hatched larvae on top of the diet [29]. The artificial diet consisted of dried yeast (7.93% *w*/*w*), wheat germ (7.93% *w*/*w*), cane sugar (7.93% *w*/*w*), corncob powder (11.89% *w*/*w*), sodium benzoate (0.47% *w*/*w*), citric acid (0.40% *w*/*w*), and water (63.44% *w*/*w*) (modified from [29] in that hydrochloric acid and the vitaminic supplement originally reported were no longer used, and citric acid is now used in the formulation). Trays with diet and larvae were covered with pieces of pantyhose fabric and incubated in a dark laboratory at 29 ± 1 °C and 70 ± 5% RH [29]. After nine days, larvae were collected from the diet by washing the contents of the trays with tap water through a plastic strainer. At this stage, larvae were used to measure the various morphological, life-history, and chemical traits considered as response variables in the experimental design (for details see Section 2.3 Experimental Design, and 2.4 Experimental Procedures and Estimation of Variables).

### 2.2. Anastrepha ludens Orange Bagasse Diet-Rearing System

This colony started in 2016 with a batch of 1000 first-instar larvae of *A. ludens* from the artificial diet-rearing system. The orange bagasse consisted of the waste of the orange fruit after it was squeezed to obtain juice and was acquired at no cost from local juice bars and cafeterias in Xalapa and Xico, Veracruz, Mexico. Orange bagasse comprises the peel/rind, remnants of juice vesicles, and the white membrane that separates the pulp segments and forms most of the endocarp. The rind is formed by the exocarp or flavedo and the mesocarp or albedo [35,36]. Each half of the bagasse of an orange fruit was minced into 6–8 pieces. Two hundred grams of pieces of orange bagasse were hand-mixed with 1 g of sodium benzoate. A layer (100 g) of the orange bagasse was then placed in a plastic container (11 cm in diameter × 7 cm in height) and pressed by hand against the bottom of the container. A piece of moistened terylene cloth with 1000 *A. ludens* first instar larvae from the artificial diet-rearing system was placed on top of the bagasse, then, the rest (100 g) of the orange bagasse was used to cover the cloth with larvae. The container with diet and larvae (i.e., the rearing container) was covered with a piece of pantyhose fabric and incubated under the same conditions, as described for the artificial diet-rearing system: continuous darkness at 29 ± 1 °C and 70 ± 5% RH. The orange bagasse diet with larvae was moistened with a 0.2% (*w*/*v*) sodium benzoate solution to maintain the humidity of the rearing substrate and prevent the growth of microorganisms. The rearing container was inspected every other day to visually confirm larval feeding activity and development. Seventeen days after the larvae were inoculated onto the orange bagasse diet, we found the first pupa, which was retrieved directly from the rearing container and moved to a laboratory at 26 ± 1 °C, 60 ± 5% RH, and 12: 12 h L: D photoperiod. Twenty days after the 1000 larvae were inoculated to the orange bagasse diet, we recovered a total of 90 pupae, from which 26 females and 16 males emerged. These 42 adults were the founders of the colony reared on the orange bagasse diet.

Adults were held in a plexiglass cage (30 cm × 30 cm × 30 cm), with ad libitum access to water and food (a 3:1 mixture of sugar: hydrolyzed protein) at 26 ± 1 °C, 60 ± 5% RH, and 12: 12 h L: D photoperiod. As in the artificial diet-rearing system, adult flies from the orange bagasse diet reached sexual maturity and started to mate and lay eggs ca. 10 days after emergence. Female flies oviposited into circular oviposition devices, as described before for the artificial diet-rearing system. Eggs were collected for ten consecutive days and incubated in the same conditions, as described before for the artificial diet-rearing system (i.e., in a Petri dish with moistened felt and a piece of terylene cloth in a 24 h dark laboratory at 29 ± 1 °C and 70 ± 5% RH). The first collections of eggs from the founder population had a volume that ranged between 0.01 and 0.5 mL. The mean (± SE) number of eggs in 0.1 mL was 1373 (± 69) (*n* = 10), and the mean (± SE) egg hatch after four days of incubation was 73.1 ± 2.71 (*n* = 10 samples of 100 eggs each). All the eggs from each day of collection were inoculated into a fixed amount of 200 g of diet, as described before, but this time the dimensions of the containers were 9.1 cm diameter × 7.7 cm height. The orange bagasse diet with larvae was sprayed with a 0.2% sodium benzoate solution. The rearing container was inspected every other day to confirm larval activity and pupation. Using this system, pupation started ca. 24 days after the larvae were inoculated onto the diet (i.e., larval development time was ca. 24 days) and pupae were collected for three consecutive days. When adult flies emerged, they were placed in a plexiglass cage, as described above, and the cycle was repeated. After 10 generations of constant rearing on the orange bagasse diet, we started to use 1 kg of orange bagasse diet inoculated with a fixed volume of 0.1 mL of eggs/hatching larvae. This volume of eggs inoculated into the diets was based on preliminary tests, suggesting that a volume of eggs between 0.062 and 0.125 mL inoculated into 1 kg of diet would produce individuals with higher pupal weights and higher egg-to-adult transformation than higher volumes of eggs (in the range of 0.125–0.25 mL) inoculated into the same amount of diet (Table A1).

In this study, we used 1 kg of orange bagasse diet inoculated with 0.1 mL of eggs (equivalent to ca. 1000 larvae) of flies from generation F25, and from generation F42 for sexual behavior assay. The cloth with eggs/larvae was placed in a thin slice (ca. 1 cm thick) of fresh sweet orange, and then the slice with larvae was covered with orange bagasse in rearing containers (39 cm long × 29 cm wide × 13 cm high). The containers with diet and larvae were incubated in a 24 h dark laboratory at 29 ± 1 °C and 70 ± 5% RH. The diets were sprayed every other day with the 0.2% sodium benzoate solution. After 13 days, the contents of the rearing containers were emptied into perforated plastic baskets (28 cm long × 23 cm wide × 17 cm high) that were placed inside a larger container (31 cm long × 25 cm wide × 8 cm high) with 10 g of vermiculite as a pupation substrate. These containers were covered with a piece of pantyhose fabric. After 24 h, the perforated basket with diet and larvae was sifted to allow the larvae to fall into the container with vermiculite. Then, the larvae collected from each rearing container were placed in a 150 mL container with vermiculite in a pupation laboratory at 22 ± 1 °C and 75 ± 5% RH. Pupation was checked every 24 h over a 72-h period after the separation of larvae from the diet. All the pupae recovered at this point were used as described in Section 2.4. Experimental Procedures and Estimation of Variables.

### 2.3. Experimental Design

The study was divided into two experimental phases. First, the explanatory variable was the diet-rearing system with two levels: (i) artificial diet and (ii) orange bagasse diet. Response variables were based on standard production and quality control parameters [37] adapted to laboratory rearing conditions for *A. ludens* [14,38], namely: (i) the number of larvae recovered; (ii) percentage of pupation; (iii) pupal yield (number of pupae per g of diet); (iv) mean pupal weight (mg); (v) the percentage of adult emergence; (vi) mean time to emergence (days); (vii) protein content in the diet (%); (viii) protein content in larvae (%); (ix) the profile of chemical compounds identified in the volatiles of sexually mature male flies (number of compounds, and their concentration [µM] in the case of compounds confirmed with authentic standards). The goal of the design was to examine the effects of the diet-rearing system on the response variables and investigate differences between populations of flies established on the artificial diet-rearing system and the orange bagasse diet-rearing system. The experimental unit varied depending on the response variable that was measured. To measure the number of larvae recovered per rearing tray (*n* = 20), pupal yields (pupae/g of diet) (*n* = 20), the protein content in the diet, and larvae (%) (*n* = 5), in each case, the experimental unit was a rearing tray (23 cm long × 18 cm wide × 12 cm high) with diet and larvae, as described for the artificial diet and orange bagasse diet rearing systems. To measure the percentage of pupation (*n* = 20), the experimental unit was a pupation tray (23 cm long × 18 cm wide × 12 cm high) with vermiculite and third-instar pupating larvae recovered from each diet. To measure the mean pupal weight (mg) (*n* = 20), the experimental unit was a pupal container (9 cm of diameter × 7.5 cm high) with pupae recovered from each diet. To measure the percentage of adult emergence (*n* = 20) and the mean time to emergence (days) (*n* = 20), the experimental unit was a plastic dish (10 cm × 10 cm) with 100 individualized pupae (*n* = 20). To measure the number of chemical compounds identified in the volatiles of sexually mature male flies and the concentration (µM) of the compounds confirmed with authentic standards (*n* = 16), the experimental unit was a glass jar with 15 sexually mature *A. ludens* males. Details on the experimental procedures and how the variables were measured are given in Section 2.4. Experimental Procedures and Estimation of Variables. The experiment was performed on 14 different days, with one or two replicates per rearing system per day.

In the second experimental phase, we examined the sexual competitiveness and aspects of the sexual behavior of males from the orange bagasse diet-rearing system, and we compared them to males from the artificial diet-rearing system and from the wild host *C. edulis* (White Sapote). The explanatory variable of the design was the origin of male flies with three levels: (i) orange bagasse diet, (ii) artificial diet, and (iii) White Sapote. Response variables were: (i) the proportion of copulations, (ii) the time to start copulation (min), and (iii) copula duration (min). The experimental arena was a cage with potted orange trees (mimicking the canopy of a medium-sized orange tree), sexually mature adults from each origin (i.e., orange bagasse diet, artificial diet, and White Sapote), and females that developed in White Sapote. A total of 10 replicates (cages) were run over five days. Details on the experimental procedures and how the variables were measured are found in Section 2.4.9. Sexual Competitiveness and Aspects of Sexual Behavior.

### 2.4. Experimental Procedures and Estimation of Variables

#### 2.4.1. Number of Larvae Recovered per Rearing Tray

In the case of the artificial diet-rearing system, larvae were recovered nine days after inoculation into the diet by washing the contents of the rearing tray with tap water through a plastic strainer. The recovered larvae were dried by placing them in a plastic tray (32 cm long × 19 cm wide ×14 cm height) with absorbent towels (Scott Shop towel, Scott Brand, Kimberly-Clark Corp., Neenah, WI, USA). Once dried, the volume of all the larvae recovered per tray was measured with a graduated cylinder. The number of larvae in a 5 mL sample was counted, and the number of larvae per tray was estimated as the volume (mL) of all the larvae in a tray multiplied by the number of larvae in a 5 mL sample divided by five. Then, larvae were placed in the same tray as before and mixed with vermiculite in a 2:1 vermiculite: larvae ratio. Trays with larvae in vermiculite were placed in a pupation laboratory at 22 ± 1 °C, 70 ± 5% RH, and 12: 12 h L: D photoperiod.

In the case of the orange bagasse-rearing system, larvae were recovered 13 days after inoculation into the diet by placing the contents of rearing containers into perforated plastic baskets (28 cm long × 23 cm wide × 17 cm high), which were placed inside larger containers (31 cm long × 25 cm wide × 8 cm high) with 10 g of vermiculite as a pupation substrate, as described before. These containers were covered with a piece of pantyhose. After 24 h, the perforated basket with diet and larvae was sifted so that the larvae fell into the container with vermiculite. The larvae were counted directly in cases where there were few individuals, or the number of larvae was estimated indirectly by measuring a specific volume of larvae and counting the number of larvae in a reference volume, as explained for larval counts in the artificial diet-rearing system. The larvae were placed in 150 mL containers with vermiculite in a pupation laboratory at 22 ± 1 °C and 75 ± 5% RH.

#### 2.4.2. Pupation Percentage

Pupation was checked 24 h after larval separation from diets in the case of the artificial diet-rearing system. In the case of the orange bagasse diet-rearing system, pupation was checked every 24 h over a 72-h period. The percentage of pupation was estimated by dividing the number of pupae found per tray by the total number of larvae in the same tray, and the resulting quotient was multiplied by 100. Pupae were placed in plastic containers (9 cm diameter by 7.5 cm height) in a laboratory at 27 ± 1 °C, 63 ± 5% RH, and 12: 12 h L: D photoperiod for further evaluations of pupal weight and adult emergence.

#### 2.4.3. Pupal Yield

The volume of pupae produced per tray was measured with a graduated cylinder, and the number of pupae in a 5 mL sample was counted. The number of pupae per tray was estimated as the volume (mL) of all the pupae in a tray multiplied by the number of pupae in a 5 mL sample, divided by five. The yield of pupae per tray was estimated as the number of pupae divided by the mass (g) of the diet used (1200 g in the case of the artificial diet and 1000 g in the case of the orange bagasse diet).

#### 2.4.4. Pupal Weight

The pupae recovered from each rearing tray were moved to a laboratory with a 12: 12 h L: D photoperiod at 27 ± 1 °C and 63 ± 5% RH. When pupae reached three days of age, samples of 100 pupae were weighed on an analytical balance (Citizen CX 220, Citizen Pvt. Ltd., Hermle, Germany). In the case of the orange bagasse diet-rearing system, the 100 pupae sample included pupae from the 24, 48, and 72 h collections. The mean pupal weight was estimated for each experimental unit. After being weighed, the samples of 100 pupae were placed in individual cells (1.6 × 1.6 cm) of a plastic dish with an acrylic lid in a laboratory with a 12: 12 h L: D photoperiod at 27 ± 1 °C and 63 ± 5% RH to determine adult emergence.

#### 2.4.5. Adult Emergence

Fifteen days after pupation, adult emergence was checked every 24 h over a 72-h period. The percentage of adult emergence from each experimental unit (i.e., a plastic dish with individualized cells and an acrylic lid as described before) was calculated by multiplying the number of adults that emerged by 100, and the product was divided by the number of pupae placed in each plastic dish with individual cells (i.e., 100 pupae).

#### 2.4.6. Time to Emergence

Time to emergence was estimated as the average time in days that elapsed from the day of pupation to the day of adult emergence (*n* = 100 pupae, the same pupae used to estimate adult emergence).

#### 2.4.7. Protein Content in Larvae and Diets

Third instar larvae were collected at nine days in the case of the artificial diet and at 12–13 days in the orange bagasse diet. Larvae were collected from the diets with soft entomological forceps, washed with distilled water, and dried by placing them on absorbent paper towels. The larvae were placed in Petri dishes and left for 24 h at 29 °C and 70% RH to let them evacuate all diet from their gut. The larvae with a clean gut (confirmed by visual inspection of the larvae) were placed in a sterile 15 mL falcon tube and frozen at −80 °C. Frozen larvae were freeze-dried (Labconco Freezone 1 Freeze Dry System, Labconco Corp., Kansas City, MO, USA) for four days and then ground in a porcelain mortar with a pestle. Samples consisted of 2 g of larvae from each diet (ca. 98 larvae in the case of the orange bagasse diet and 74 larvae in the case of the artificial diet).

In the case of the artificial diet and the orange bagasse diet, we used 50 g samples of freshly prepared diet in sterile 15 mL falcon tubes. Samples of freshly prepared diet were frozen at −80 °C for 24 h and then lyophilized (Labconco, Freezone 1) for three days. Samples were then ground in a porcelain mortar with a pestle.

The protein content of the samples (larvae and diets) was determined by the Dumas method [39] using an elemental analyzer (CHN model 2400, Perkin Elmer, NJ, USA). The protein content was estimated by multiplying the nitrogen content by a constant value of 6.25 [39]. Five independent samples of 2 ± 0.1 mg (dry weight) of larvae and diets were used for the analyses.

#### 2.4.8. Chemical Compounds Identified in the Volatiles of Sexually Mature Male Flies

- Male Odor Collections

Newly emerged flies from each treatment were released inside acrylic cages (20 × 20 × 20 cm) and were separated by sex by capturing them with a glass vial and then transferring them into plastic containers (12 cm long × 13 cm wide × 13 cm high) with food (a 3: 1 mixture of sugar: hydrolyzed protein) and water ad libitum. Groups of 25 females or males remained in their cages until they reached sexual maturity. The dynamic aeration technique [40] was used to collect volatile compounds from sexually mature virgin males (15–18 days of age). Sixteen replicates for each fly origin were performed, and each one had a control treatment (clean air in an empty chamber), as explained below.

Fifteen *A. ludens* males of the same age and larval diet treatment were placed inside a collection chamber (12.4 cm high × 8.4 cm diameter glass jar with a lid adapted for air inlet and outlet). A continuous flow of purified air (1 L/min) was injected into the chambers to mix with the male odors and then extracted at the same air flow with a vacuum system connected to the end of the odor trap (VCT-1/4-3-HSQ-P, ARS), containing an odor adsorbing material (Super Q). Considering that *A. ludens* males display courtship behavior in the afternoon, the volatile collection time was two hours, from 3:00 to 5:00 pm. Volatile compounds emitted by males were recovered from the adsorbent material by eluting with 400 μL of dichloromethane (Sigma-Aldrich, Saint Louis, MO, USA, HPLC grade) and were stored in 2 mL amber glass vials at −80 °C until their chromatographic analyses.

- Chemical Analyses of Male Odors

Volatile compounds were analyzed by electron impact gas chromatography-mass spectrometry (GC-MS) by using a gas chromatograph (2010 Plus, Shimadzu Inc., Kyoto, Japan) fitted with a ZB-5MSI column (30 m, 0.25 mm i.d., 0.25 μm film thickness) and coupled to a MS detector (QP-2010 Ultrasystem, Shimadzu Inc.), as described by Aluja et al. [12]. Samples (1 µL) were injected into the GC injector at 250 °C in splitless mode. The GC oven was programmed as follows: 50 °C for 5 min, then a ramp of 15 °C/min up to 280 °C for 5 min. A solvent delay time of 3 min was used. Helium was used as the carrier gas at a 1 mL/min flow rate. The MS ion source temperature was 200 °C, and the interface was 250 °C. For tentative identification, we compared the mass spectra of the compounds against those recorded in the NIST (National Institute of Standards and Technology, Gaithersburg, MD, USA) library, version 2.0D, NIST/EPA/NIH (NIST05). For qualitative analysis, the abundances (counts) of the compounds reproducible in more than 80% of the replicates were considered. Confirmation was achieved by using authentic standards when they were available. Quantification of (*Z*)-3-nonen-1-ol, (*Z,Z*)-3,6-nonadien-1-ol (Sigma Aldrich, Saint Louis, MO, USA, HPLC grade), (*E*,*E*)-α-farnesene (Toronto Research Chemicals, TRC-Canada, Toronto, ON, Canada), anastrephin, and epianastrephin (USDA) was performed by using a calibration curve with six concentrations of authentic standards and adjusted to a linear regression (r^2^ ≥ 0.99).

#### 2.4.9. Sexual Competitiveness and Aspects of Sexual Behavior

- Fly Origin and Handling

The wild flies originated from naturally infested *C. edulis* fruit collected in Guadalcazar, San Luis Potosí, Mexico (22°37′7.55″ N, 100°23′45.57″ W, 1658 m elevation), transported by car to our laboratories in Xalapa, Veracruz (ca. 785 km from Guadalcazar) inside of plastic trays with vermiculite. In a laboratory at room temperature (23 °C), fruits were placed in 31 × 25 × 13 cm plastic racks, which, in turn, were placed on top of 32 × 19 × 14 cm plastic washbowls, containing a layer of vermiculite as a pupation medium. Larvae were allowed to exit freely from the fruit and exit through the holes in the plastic rack into the vermiculite to pupate. Every 24 h, we searched for pupae in the vermiculite and transferred them to a 150 mL plastic container with 15 mL of vermiculite. The vermiculite was regularly moistened with a 0.2% (*w*/*v*) sodium benzoate solution. Pupae of all treatments were kept in a laboratory at 27 ± 1 °C, 63 ± 5% RH, and 12: 12 h L: D photoperiod. About 15 days after pupation, adult flies started to emerge. At this moment, flies were separated by sex and placed in a 20 cm (length) × 13.5 cm (height) × 13.5 cm (width) plastic cage with ample aeration (walls were covered with an organdy mesh) and provided with food (3: 1 mixture of sugar and hydrolyzed protein) and water ad libitum. We placed 20 adults in each cage and kept them until they reached sexual maturity (14 days of age).

- Marking of Males

**Figure 1:**
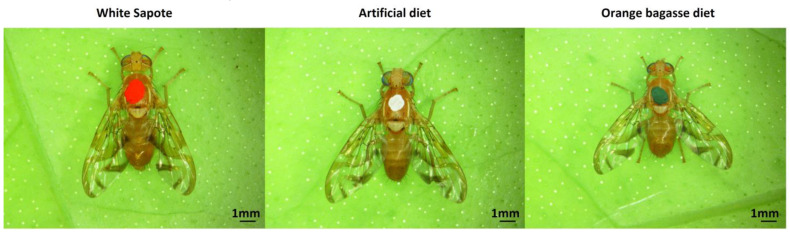
Marked *A. ludens* males from different origins used in the sexual competitiveness test.

When adults (independent of dietary origin) reached 14 days of age, they were marked in the thorax with a visible dot of watercolor (Vinci, Estado de Mexico, Mexico) 24 h before using them in the sexual behavior test. All males from the same diet/origin were marked with the same color, but colors were interchanged every day to avoid a possible bias on female selectivity depending on color. The colors used were green, red, and white (Figure 1). We marked 15 males per color every day, of which five were used per treatment in each observation cage (ten males in total), and five were maintained as replacements in case a male died (i.e., a total of 45 males of the three colors used every day were marked). All marked males were kept in the same type of cages described before, but they were separated by age (we only used 14-day-old males and females in all the tests).

- Laboratory Sexual Behavior Test

**Figure 2:**
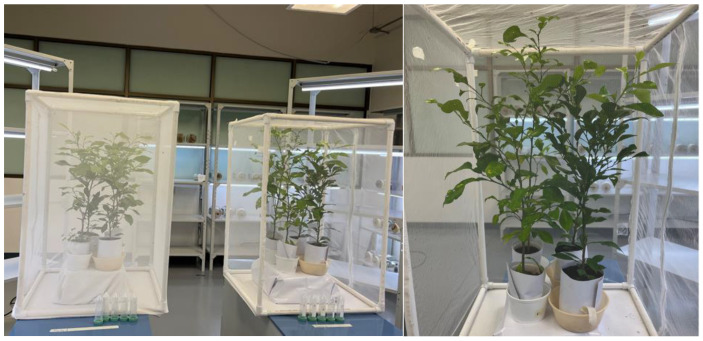
The laboratory setting and experimental cages to study the sexual behavior of *A. ludens* adults from different larval diets (artificial diet, orange bagasse diet and White Sapote, *C. edulis*).

The test consisted in releasing five males of each origin (i.e., wild flies and flies reared in the orange bagasse and artificial diets) in a 60 cm length × 60 cm width × 90 cm height cage covered with a nylon mesh to allow for aeration, light penetration and observations (Figure 2), into which four potted orange trees (ca. 70 cm tall and canopy width of 40 cm) had been introduced to offer males perches (leaves/branchlets) from which to display, as males call with their wings and emit sexual pheromones that are attractive to females [5,41]. We also released five wild females (originating from *C. edulis*). So, there were fifteen males and five females in the experimental arena when the experiment started. The same morning of the test, males were released into the cage at 10:00 am, and females were released at 5:00 pm (ca. 15–30 min before males started to call). We observed all male and female sexual activity continuously from 5:00 to 7:30 pm, or until the last copulating pair separated. We ran two simultaneous tests (cages) (Figure 2) on each day, with two observers assigned to each cage. The variables measured were: (i) the time of initiation/end of copulation and (ii) the paint dot color of the male involved.

To simulate natural conditions (*A. ludens* is a crepuscular mating species [5]), we adjusted luminosity in the laboratory every hour until total darkness. Tests started with a luminosity of 443 lux at 5:00 pm, which was reduced to 87, 41, and 4 lux, each hour, until we reached total darkness (0 lux). All copulations started when there was still a certain degree of light in the laboratory, so we could identify the color of the male that was involved in a particular mating. Once it became dark, we cautiously used the light of a cellular phone (i.e., lit the copulating pair sideways so the light did not disturb the flies) to ensure we recorded the exact moment when the pair disengaged. Tests were performed at 27 ± 1 °C and 70 ± 5% RH.

### 2.5. Statistical Analyses

In the first experimental phase, we used two-sample *t*-tests to compare the mean values of the response variables (production and biological traits of *A. ludens*, the chemical compounds identified in the volatiles of sexually mature calling *A. ludens* males, and the protein content in diets and larvae from each diet) and the 95% confidence interval (CI) of their differences as a function of the explanatory variable (rearing system with two levels: artificial diet and orange bagasse diet). The differences in means and their 95% CIs were used to illustrate the magnitude and uncertainty in the differences between the production and traits of flies reared on the artificial diet-rearing system and the orange bagasse diet-rearing system [42]. We used *t*-tests for equal variances (in the cases of pupation, protein content in diet and larvae, concentrations of: (*E*,*E*)-alpha-farnesene, epianastrephin, (*Z*,*Z*)-3,6-nonadien-1-ol and anastrephin) and unequal variances (in the cases of the number of larvae recovered, yield, pupal weight, adult emergence, time to emergence, and concentration of (*Z*)-3-nonen-1-ol). In the case of the chemical compounds in the volatiles of sexually mature males, we present qualitative and semi-quantitative data in a hierarchical clustering heatmap using peak areas Log (base 10) transformed data, Euclidean distance, and the Ward.D clustering algorithm in MetaboAnalyst (Wishart Research Group, Edmonton, AB, Canada) 5.0, a web-based bioinformatic platform [43].

In the second experimental phase, we used linear models to estimate the mean values and 95% CIs of the proportion of mating (number of matings of adults from each origin divided by the total number of matings per cage), the time to start mating (min), and the copula duration (min) as a function of the origin of male flies.

We report results in terms of statistical clarity [44] and language of evidence [45]. We used R [46] and RStudio (Boston, MA, USA) [47] for *t*-tests and linear models.

## 3. Results

**Figure 3:**
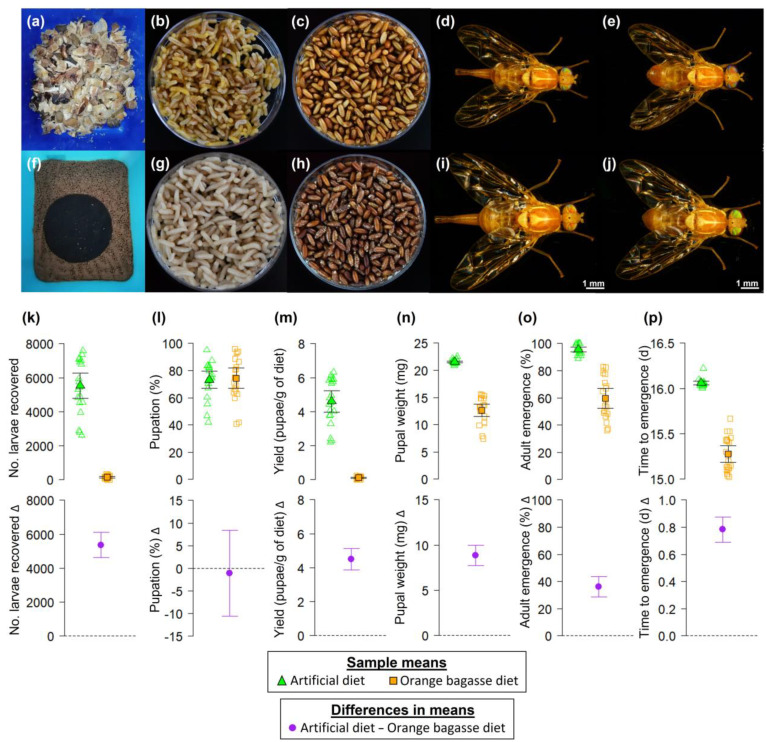
*Anastrepha ludens* flies reared on an orange bagasse diet (**a**–**e**) and an artificial diet (**f**–**j**). Note the orangish coloration of the larvae and pupae from the orange bagasse diet (**b**,**c**) compared to the larvae and pupae from the artificial diet (**g**,**h**), as well as the smaller size of adults from the orange bagasse diet (**d**,**e**) compared to those from the artificial diet (**i**,**j**). (**k**–**p**) Production and biological traits of *A. ludens* from an artificial diet-rearing system and an orange bagasse diet-rearing system; the upper row shows data points (open symbols) and sample means (solid symbols). Error bars indicate 95% confidence intervals (95% CI). The bottom row shows differences in means; 95% CI of the difference that does not include zero indicates statistically significant differences between means.

After 24 generations of constant rearing on a diet based on orange fruit bagasse, flies from this colony were physically characterized by larvae and pupae of an orangish coloration and adults of smaller size when compared to individuals reared on the artificial diet (Figure 3a–j). We found clear statistical differences between the numbers of larvae recovered from the artificial diet-rearing system and the orange bagasse diet-rearing system (*t* = 14.85, df = 19.15, *p*-value = 5.813 × 10^−12^, Figure 3k). On average, 5370 more larvae were obtained from each tray of artificial diet compared to the larvae obtained from the orange bagasse diet (Figure 3k). The effect of the rearing system on the mean percentage of pupation was statistically unclear (*t* = 0.24, df = 38, *p*-value = 0.811; Figure 3l). On average, 4.49 more pupae were obtained per g of the artificial diet than per g of the orange bagasse diet (*t* = 14.91, df = 19.11, *p*-value = 5.571 × 10^−12^, Figure 3m). The artificial diet produced pupae that weighed, on average, 8.87 mg more than the pupae from the orange bagasse diet (*t* = 16.36, df = 20.05, *p*-value = 4.572 × 10^−13^, Figure 3n). The mean adult emergence of individuals from the artificial diet-rearing system was 36.08% higher than that of individuals from the orange bagasse diet-rearing system (*t* = 10.10, df = 21.19, *p*-value = 1.488 × 10^−9^, Figure 3o). Flies reared on the artificial diet took 0.783 more days to emerge as adults than flies reared on the orange bagasse diet (*t* = 17.67, df = 20.99, *p*-value = 4.431 × 10^−14^, Figure 3p).

**Figure 4:**
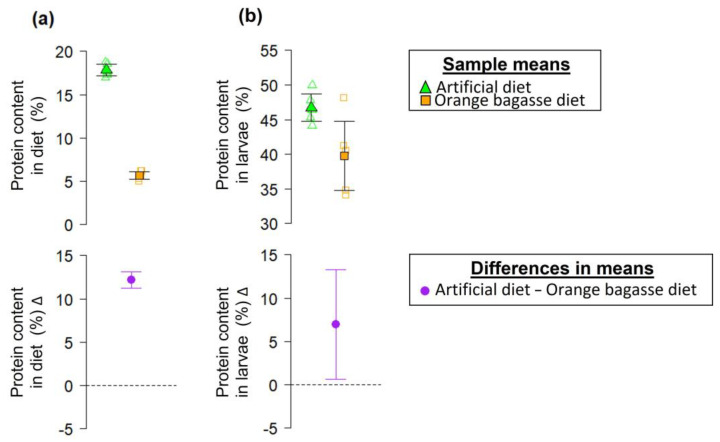
The protein content (% of dry weight) in (**a**) the artificial and orange bagasse diets and (**b**) in third instar *A. ludens* larvae reared on either the artificial diet or the orange bagasse diet. The upper row shows data points (open symbols) and sample means (solid symbols). The bottom row shows differences in means. Error bars indicate 95% confidence interval (95% CI). In the bottom row, 95% CI of the difference that does not include zero indicates statistically significant differences between means.

The difference between the protein content in the artificial diet and the orange bagasse diet was statistically clear (*t* = 29.85, df = 8, *p*-value = 1.72 × 10^−9^). On average, the artificial diet had 12.18% more protein than the orange bagasse diet (Figure 4a). The difference between the protein content of larvae from the artificial diet-rearing system and the orange bagasse diet-rearing system was statistically clear (*t* = 2.53, df = 8, *p*-value = 0.035). Larvae from the artificial diet-rearing system had an average of 6.94% more protein content than larvae from the orange bagasse diet-rearing system (Figure 4b). It is noteworthy that, somehow, larvae were able to accrue enough protein from the bagasse, as the difference in protein content in larvae originating from this substrate compared with those from the artificial diet was much smaller than the difference in protein content between the artificial diet and the orange bagasse.

**Figure 5:**
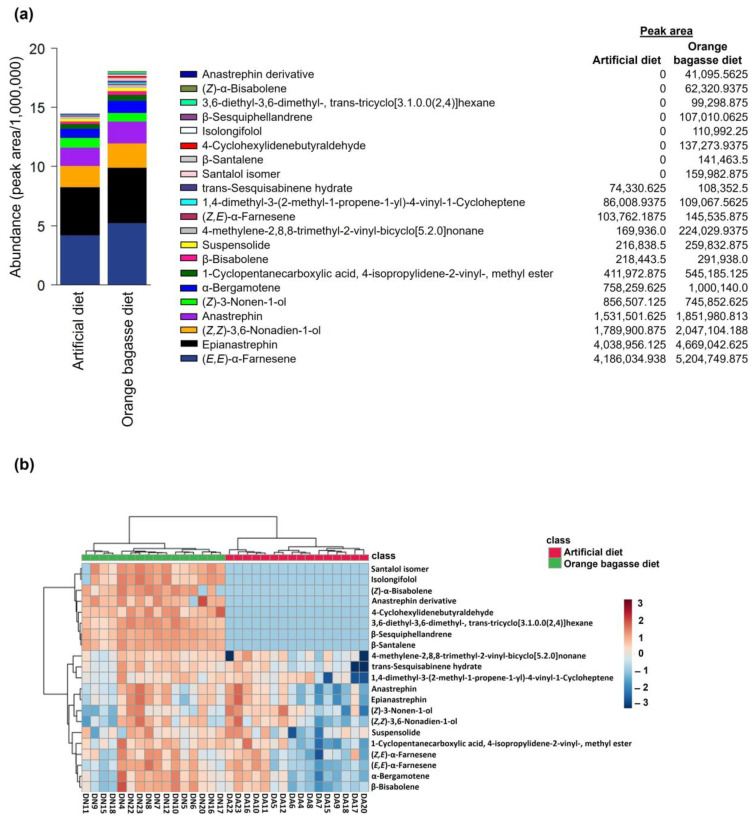
Chemical compounds tentatively identified (by comparing mass spectra registered in the National Institute of Standards and Technology [NIST] library or confirmed with authentic standards when they were available) in the volatiles of sexually mature calling *A. ludens* males that developed in either an artificial diet or an orange bagasse diet. (**a**) The abundance (peak area/1,000,000) of the 21 compounds tentatively identified. (**b**) Heatmap showing the clustering result of tentatively identified chemical compound abundances (peak areas Log transformed) in male flies from artificial and orange bagasse diets. The red–blue color scale corresponds to Z-score values that were calculated as the abundance value of each compound minus the population mean divided by the population standard deviation.

We found 13 chemical compounds in the volatiles of sexually mature *A. ludens* males that developed as larvae on the artificial diet, whereas adults that developed in the orange bagasse diet had the same 13 compounds, plus eight additional compounds not found in adults from the artificial diet (Figure 5). Regardless of the larval diet-rearing system, the most abundant compounds in the volatiles of sexually mature *A. ludens* males were (*E*,*E*) -α-farnesene and epinastrephin, followed by (*Z*,*Z*)-3,6-nonadien-1-ol, anastrephin, and (*Z*)-3-nonen-1-ol (Figure 5a). The clustering analysis of the abundances of all tentatively identified compounds in the scent bouquet of males grouped the males from the artificial diet and separated them from those of the orange bagasse diet (Figure 5b). Two main groups of compounds were observed; one included the eight compounds found exclusively in males from the orange bagasse diet, and the second group clustered common compounds of males from both diet origins (Figure 5b).

**Figure 6:**
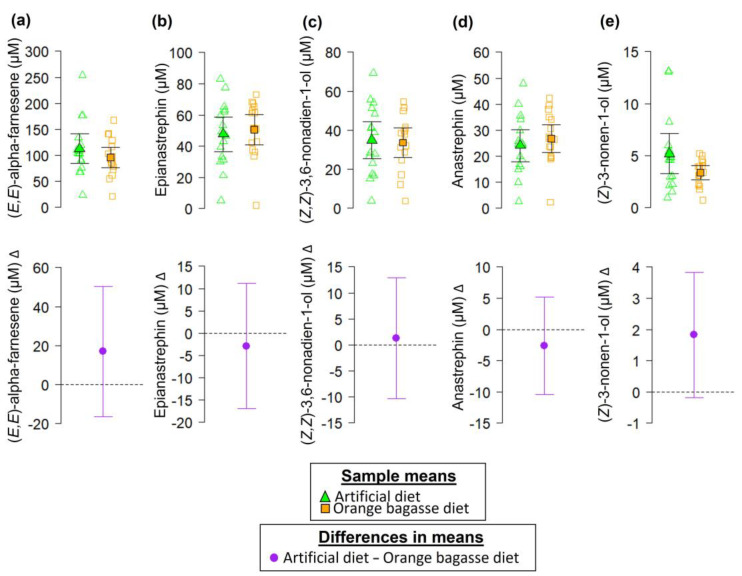
Chemical compounds identified and confirmed with authentic standards in the volatiles of sexually mature *A. ludens* males developed in either an artificial diet or an orange bagasse diet. Panels (**a**–**e**), show the compounds (*E*,*E*)-alpha-farnesene, epianastrephin, (*Z*,*Z*)-3,6-nonadien-1-ol, anastrephin, and (*Z*)-3-nonen-1-ol, respectively. The upper row shows data points (open symbols) and sample means (solid symbols) of the compounds confirmed with authentic standards as a function of the diet in which larvae develop; the bottom row shows differences in means. Error bars indicate 95% confidence interval (95% CI). In the bottom row, the lack of a significant difference between means is denoted by a 95% CI that includes zero.

Our analyses did not reveal statistically clear effects of the larval diet on the concentration of the compounds confirmed with authentic standards: (*E*,*E*)-alpha-farnesene (*t* = 1.04, df = 30, *p*-value = 0.3048; Figure 6a), epianastrephin (*t* = 0.42, df = 30, *p*-value = 0.6798; Figure 6b), (*Z*,*Z*)-3,6-nonadien-1-ol (*t* = 0.23, df = 30, *p*-value = 0.817; Figure 6c), anastrephin (*t* = 0.69, df = 30, *p*-value = 0.4965; Figure 6d), and (*Z*)-3-nonen-1-ol (*t* = 1.91, df = 18.62, *p*-value = 0.0715; Figure 6e).

**Figure 7:**
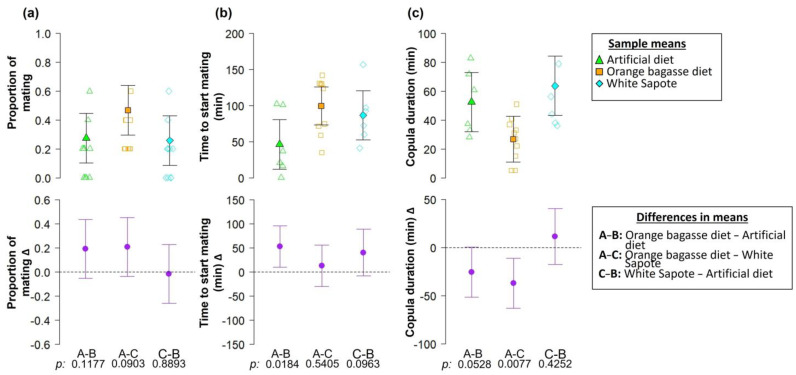
(**a**) The proportion of mating, (**b**) time to start mating, and (**c**) copula duration of *A. ludens* explained by the origin of male flies (artificial diet, orange bagasse diet, and White Sapote). The upper row shows data points (open symbols) and sample means (solid symbols); the bottom row shows differences in means. Error bars indicate 95% confidence interval (95% CI). In the bottom row, the lack of a significant difference between means is denoted by a 95% CI that includes zero.

The proportion of matings gained by males from the orange bagasse diet was approximately 20% higher than that observed in males from the artificial diet and from White Sapote (*C. edulis*), but we were unable to find statistically clear evidence of a relationship between the larval diet and the proportion of matings gained by males (*F* = 1.9; df = 2, 27; *p*-value = 0.1681; Figure 7a). The number of matings of males from the artificial diet, White Sapote, and the orange bagasse diet ranged from one to three (Figure 7a).

We found weak statistical evidence pointing to an effect of the larval diet on the time when male flies started mating (*F* = 3.4; df = 2, 19; *p*-value = 0.0548; Figure 7b). On average, males from the artificial diet started mating 53.3 and 40.4 min earlier than males from the orange bagasse diet and White Sapote (*C. edulis*), respectively (Figure 7b).

We found a clear difference in copula duration of males from the different larval diets (*F* = 4.9; df = 2, 19; *p*-value = 0.0185; Figure 7c). On average, the longest copulations were found in males from White Sapote (*C. edulis*) (60.19 min), followed by males from the artificial diet (50.30 min) (Figure 7c). The shortest individual copulations (5 min) were observed in males from the orange bagasse diet (Figure 7c). On average, copula duration of males from the orange bagasse diet was 25.70 and 37.02 min shorter than the copulas of males from the artificial diet and White Sapote (*C. edulis*), respectively (Figure 7c).

## 4. Discussion

We report the development of a rearing system for the frugivorous fly *A. ludens* on a larval diet based exclusively on organic residues, specifically orange bagasse. The adaptation of *A. ludens* to the nutritionally poor orange bagasse diet and the changes in the life-history (emergence, time to emergence), morphological (pupal body mass/weight), nutritional (protein content in larvae), and chemical (odor composition of sexually mature calling males) traits of flies compared to individuals from the artificial diet-rearing system (Figure 3, Figure 4, Figure 5 and Figure 6) might reflect a case of phenotypic plasticity in response to a stressful larval developmental environment [16,48]. In addition to the low protein content of the orange bagasse, the chemical composition of the rind of the orange bagasse is complex, including sugars, minerals (e.g., iron, manganese, calcium), vitamins (e.g., vitamin C, provitamin A, folate, riboflavin, thiamine, vitamin B6), flavonoids, such as naringin, hesperidin, neohesperidin, nariratin, didymin, eriocitrin (flavonones), diomin, lateolin, sinensetin, nobi-letin (flavones), and phytoene, β-crytoxanthin, and violaxantin (carotenoids), terpenoids (limonene and linalool), alkaloids, tannins, saponins, resins, and esters [31,49,50,51,52,53]. As such, the *A*. *ludens* larvae adapted to the orange bagasse diet must deal with many potentially deleterious phytochemicals, plus a very low protein content, and this could partly explain their small size and the delay observed in their life cycle (i.e., the time to emergence). Additionally, our results might be explained by considering genetic bottleneck effects or inbreeding depression because of the low number of founders (42 adult flies) used to establish the orange bagasse diet colony [54].

Our results provide support to our working hypothesis that the nutritional deficiencies of the orange bagasse result in reduced fitness of flies. As we predicted, the orange bagasse diet and larvae from this diet had significantly lower protein levels than observed in insects from the artificial diet (Figure 4). It is remarkable, however, that larvae developed on the orange bagasse diet, with a three-fold lower protein content than the artificial diet, were able to efficiently metabolize proteins to attain a mean protein content of ca. 40% (Figure 4). The ability of *A. ludens* larvae to adapt to the orange bagasse diet and assimilate protein from such a relatively nutritionally poor substrate might be related to the gut microbiota or factors present in the diet [55]. In fact, the microbial communities in tephritid diets are a key component of their quality, as they can be a direct source of nutrients or facilitate the use of inaccessible nitrogen sources [11,56,57]. Behar et al. [58,59] highlighted the role of diazotrophic enterobacteria in facilitating the acquisition of nitrogen from rotting fruit pulp. Working with guava and the medfly (*C. capitata*), they report that the dominant bacterial populations in rotting guava infested by *C*. *capitata* larvae were pectinolytic and nitrogen fixing Enterobacteriaceae. Therefore, future studies should evaluate the microbiota associated with the orange bagasse diet, as this system offers many possibilities to better understand the role of gut bacteria as sources of nitrogen in nitrogen-poor diets.

The percentage of protein in larvae from the orange bagasse diet (Figure 4b) was similar to that of other insect species used for human consumption and is higher than the protein content of raw beef from cattle [60]. Therefore, the *A. ludens* orange bagasse diet-rearing system reported here could have potential applications for the sustainable production of insect protein for use in the food and feed industries.

It was recently proposed that a “core blend” of volatile compounds is preserved in the odors of sexually mature male *A. ludens* flies with the addition of new compounds as flies adapt to new host plants/larval diets [12]. In accordance with this hypothesis we put forward with respect to the “core blend”, here we demonstrated that the odors of sexually mature males from the orange bagasse diet had the same volatile compounds as the odors of males from the artificial diet, plus eight additional compounds (Figure 5). Notably, the odor bouquet of males adapted to the orange bagasse diet had 14 more compounds than the odor bouquet of males from the wild host White Sapote (*C. edulis*), reported previously [12]. The fact that the odors of males from the artificial and the orange bagasse diets shared a “core blend” of volatile compounds at similar concentrations, but differed in the overall composition of the odor bouquet (Figure 5 and Figure 6), suggests that some of these volatiles could be acquired from nutrients or secondary compounds ingested with the larval diet [3,12]. In fact, we believe that the orangish coloration of larvae and pupae from the orange bagasse diet (Figure 3b,c) indicates that larvae are sequestering compounds from their diet for later use as precursors of volatile compounds or other semiochemicals. 

Males from the orange bagasse diet with a complex scent bouquet were initially sexually competitive (Figure 7a), but their copulations with wild females from White Sapote (*C. edulis*) were on average 50–60% shorter than those observed in males from the artificial diet and White Sapote (Figure 7c). Copula duration is related to the transfer of sperm and seminal fluid proteins, and shorter copulations may indicate low sperm transfer [61]. These results do not provide clear support to our working hypothesis that the highly chemically diverse scent bouquet of sexually mature male flies would enhance their sexual attractiveness and performance. Perhaps a chemically diverse bouquet of odors in males from the orange bagasse diet had an initial attractive effect on females (i.e., a “novelty effect”), but minutes after initiating the sexual encounter, females decided to end the copulation with unusually “perfumed” males because cryptic female choice mechanisms may enter in play, allowing females to ascertain the quality of the seminal fluids, sperm, or other male quality attributes. If a chemically complex bouquet of odors “deceives” *A. ludens* females to feel attracted to low-quality males remains to be tested, but this could have applications in the development of baits for capturing female flies in the field. Future studies should examine the effects of the volatile compounds in odors from sexually mature males on female sexual attraction. Understanding the relationships between odor cues and sexual behavior is key to improving the application of biorational pest control strategies against tephritid pests [3,12], and our study provides an example of how male flies from different larval environments and with different odor profiles compete sexually for females (Figure 7).

Most of the volatile compounds collected from males from the orange bagasse diet were present only in trace amounts and contributed to less than 5% of the whole scent bouquet, but they were detected in more than 80% of the replicates (Supplementary Table S1). Of these compounds, β-sesquiphellandrene and β-santalene were previously reported in *A. ludens* males from *Malus* × *domestica* Borkh. (Rosaceae), *Pyrus communis* L. (Rosaceae)*, Punica granatum* L. (Lythraceae), and *C.* × *paradisi* [12]. 3,6-diethyl-3,6-dimethyl tricyclo[3.1.0.0(2,4)]hexane was reported in *A. obliqua* males that developed in *Spondias mombin* L. (Anacardiaceae)*, S. purpurea* L. (Anacardiaceae)*, Solanum lycopersicum* L. (Solanaceae)*, M. indica,* and *Psidium guajava* L. (Myrtaceae) [12]. A santalol isomer was reported by Bosa et al. [62] as a volatile of *A. ludens* males from a mass-reared colony, a selected strain, and a hybrid strain. This is the first report of an anastrephin derivative (rt = 15.466 min) and isolongifolol (rt = 16.062 min) (compounds tentatively identified) as constituents of the odors emitted by sexually mature *A. ludens* males from larvae reared on orange bagasse.

Our study adds to the growing body of research on *A. ludens* rearing by reporting orange bagasse as a new larval diet for this fly. Because orange bagasse is a waste product and can be obtained at no cost, the orange bagasse diet-rearing system could also have applications in mass-rearing programs of sterile flies seeking to reduce production costs [20,21]. However, although the orange bagasse diet is suitable for the continuous rearing of *A. ludens*, we note that it is a suboptimal substrate in terms of the number of larvae recovered and pupae per g of diet, pupal weight, and adult emergence compared to the artificial diet-rearing system (Figure 3). Therefore, there is a need to optimize the orange bagasse diet-rearing system to maximize insect quality and yields. Other residues from fruit processing industries (e.g., peels and seeds) could also be tested as potential substrates for rearing *A. ludens* and other tephritid species. Our study did not consider testing orange bagasse as an additional ingredient in the artificial diet formulation (e.g., as a bulking agent), but we will do so in future studies.

As *A. ludens* is a frugivorous fly that evolved from ancestors with saprophagous feeding habits [1,7], we believe that the new orange bagasse diet-rearing system offers a unique opportunity to deepen our understanding of the feeding biology and ecology of this pest. Because, in nature, a shift in the larval diet of tephritids is linked to female oviposition behavior [1], future studies will examine whether females from the orange bagasse diet can adapt to oviposit directly into the orange bagasse and will also assess if larvae could continue developing if sodium benzoate is eliminated from the diet so that microorganisms can proliferate in the bagasse. This could resemble an ancestral tephritid fruit fly diet [1,7], and it would allow us to study the evolution from saprophagy to frugivory.

## 5. Conclusions

Our study presents a novel rearing concept for frugivorous flies using fruit waste as the only substrate for larval development. Although performed at the laboratory level, we observed an extreme case of adaptation to a novel larval environment consisting of fruit waste. Based on our results, we conclude that flies feeding on orange bagasse as the only larval diet develop morphological, chemical, nutritional, and sexual behavioral characteristics different from those of flies fed on an artificial diet or flies stemming from wild host fruit. Future research is required to test the hypothesis that a complex bouquet of odors containing many compounds beyond the core pheromonal components in male flies “deceives” females to be attracted to low-quality males. 

## Data Availability

The data presented in this study are available upon request from the corresponding author.

## Supplementary Materials

The following supporting information can be downloaded at: https://www.mdpi.com/article/10.3390/biology12050739/s1, Supplementary Table S1. Volatile compounds of sexually mature *A. ludens* males from two larval diets.

## Appendix A

**Table A1:** Mean values (and standard deviation; *n* = 3) of larval recovery, pupation, pupal weight, adult emergence, and transformation from egg to adult of *A. ludens* reared on the orange bagasse diet as a function of the volume of eggs that were inoculated into 1 kg of diet.

Volume (mL) of Eggs Inoculated into 1 kg of Diet	Third Instar Larvae Recovered per g of Diet	Pupation (%)	Pupal Weight (mg)	Adult Emergence (%)	Egg-Adult Transformation (%)
0.062	0.237 (0.081)	92.4 (11.2)	17.01 (1.15)	93.3 (0.58)	24.03 (9.24)
0.125	0.413 (0.112)	81.6 (1.6)	14.78 (2.04)	95.7 (0.58)	18.85 (5.47)
0.25	0.532 (0.252)	88.7 (8.7)	13.72 (2.46)	96.7 (4.04)	13.36 (6.66)
