# Supplementary material for: Feeding on the Fruit Waste Orange Bagasse Modifies Immature Protein Content, Body Weight, Scent Bouquet Composition, and Copula Duration in Males of a Tephritid Frugivorous Fly
Source: Biology (Basel). 2023 May 19;12(5):739. doi: 10.3390/biology12050739 (PMC10215272; doi:10.3390/biology12050739)
Supplement: Supplementary file 1 [file biology-12-00739-s001.zip › biology-2257411-supplementary.pdf]

Supplementary Table S1. Volatile compounds of sexually mature *A. ludens* males from two larval diets.

Diet	Volatile Compound	Retention time (min)	Average abundance (counts/1 million)	Reproducibility
Artificial diet	(*Z*)-3-Nonen-1-ol	11.168	0.857	16/16
	(*Z,Z*)-3,6-Nonadien-1-ol	11.2	1.790	16/16
	α-Bergamotene	14.042	0.758	16/16
	1-Cyclopentanecarboxylic acid, 4-isopropylidene-2-vinyl-, methyl ester	14.393	0.412	16/16
	(*Z,E*)-α-Farnesene	14.458	0.104	16/16
	4-methylene-2,8,8-trimethyl-2-vinyl-bicyclo[5.2.0]nonane	14.5	0.194	14/16
	(E,E)-α -Farnesene	14.587	4.186	16/16
	Suspensolide	14.63	0.217	16/16
	β-Bisabolene	14.672	0.218	16/16
	trans-Sesquisabinene hydrate	14.721	0.085	14/16
	1,4-dimethyl-3-(2-methyl-1-propene-1-yl)-4-vinyl-1-Cycloheptene	14.843	0.106	13/16
	Anastrephin	15.4	1.532	16/16
	Epianastrephin	15.506	4.039	16/16
Orange bagasse diet	(*Z*)-3-Nonen-1-ol	11.168	0.746	16/16
	(*Z,Z*)-3,6-Nonadien-1-ol	11.2	2.047	16/16
	α-Bergamotene	14.042	1.000	16/16
	β-Sesquiphellandrene	14.099	0.107	16/16
	β-Santalene	14.294	0.141	16/16
	1-Cyclopentanecarboxylic acid, 4-isopropylidene-2-vinyl-, methyl ester	14.393	0.545	16/16
	(*Z,E*)-α-Farnesene	14.458	0.146	16/16
	4-methylene-2,8,8-trimethyl-2-vinyl-bicyclo[5.2.0]nonane	14.5	0.224	16/16
	(*E,E*)-α-Farnesene	14.587	5.205	16/16
	Suspensolide	14.63	0.260	16/16
	β-Bisabolene	14.672	0.292	16/16
	trans-Sesquisabinene hydrate	14.721	0.108	16/16
	1,4-dimethyl-3-(2-methyl-1-propene-1-yl)-4-vinyl-1-Cycloheptene	14.843	0.109	16/16
	4-Cyclohexylidenebutyraldehyde	14.864	0.137	16/16
	(*Z*)-α-Bisabolene	14.926	0.071	13/16
	3,6-diethyl-3,6-dimethyl-, trans-tricyclo[3.1.0.0(2,4)]hexane	15.324	0.099	16/16
	Anastrephin	15.4	1.852	16/16
	Anastrephin derivative	15.466	0.044	15/16
	Epianastrephin	15.506	4.669	16/16
	Santalol isomer	15.957	0.171	15/16
	Isolongifolol	16.062	0.118	15/16
